# Inhibition of Norovirus GII.4 binding to HBGAs by *Sargassum fusiforme* polysaccharide

**DOI:** 10.1042/BSR20240092

**Published:** 2024-09-10

**Authors:** Yiqiang Sun, Meina Liang, Mingjiang Wu, Laijin Su

**Affiliations:** 1 College of Life and Environmental Science, Wenzhou University, Wenzhou 325035, China; 2 Zhejiang Provincial Key Laboratory for Water Environment and Marine Biological Resources Protection, Wenzhou University, Wenzhou 325035, China

**Keywords:** antiviral activity, histo-blood group antigens, norovirus virus-like particles, Sargassum fusiforme polysaccharide

## Abstract

Norovirus (NoV) is the main pathogen that causes acute gastroenteritis and brings a heavy socio-economic burden worldwide. In this study, five polysaccharide fractions, labeled pSFP-1-5, were isolated and purified from *Sargassum fusiforme* (*S. fusiforme*). *In vitro* experiments demonstrated that pSFP-5 significantly prevented the binding of type A, B and H histo-blood group antigens (HBGAs) to NoV GII.4 virus-like particles (NoV GII.4 VLPs). In addition, *in vivo* experiments revealed that pSFP-5 was effective in reducing the accumulation of NoV in oysters, indicating that pSFP-5 could reduce the risk of NoV infection from oyster consumption. The results of transmission electron microscopy showed that the appearance of NoV GII.4 VLPs changed after pSFP-5 treatment, indicating that pSFP-5 may achieve antiviral ability by altering the morphological structure of the viral particles so that they could not bind to HBGAs. The results of the present study indicate that pSFP-5 may be an effective anti-NoV substance and can be used as a potential anti-NoV drug component.

## Introduction

Norovirus (NoV) is a positive single-stranded RNA virus composed of three main open reading frames (ORFs) [1,2]. NoV is easy to mutate and has many types, which can be divided into 10 genomes and 49 genotypes [3]. NoV is the main pathogen causing acute gastroenteritis. Symptoms such as diarrhea, vomiting and abdominal colic often occur after infection. There is no specific drug at present. In addition, NoV has multiple routes of transmission and a low infectious dose, resulting in a severe global disease burden.

These diseases caused by NoV are usually associated with eating raw contaminated oysters. Precipitation or sewage discharged by ships may discharge NoV into the waters where oysters grow. As filter feeders, oysters filter a large amount of seawater through gills. Once their living waters are contaminated, oysters can concentrate and enrich a large number of viruses, which are tens or even thousands of times higher than the environmental concentration. For NoV to become non-infectious, it is necessary to keep it at 100°C for more than 15 min, which seriously destroys the freshness and nutritional value of oysters. Therefore, people usually eat raw oysters or simply cook them, which results in oysters playing a more important role in NoV transmission.

Histo-blood group antigens (HBGAs) are target receptors of NoV and have multiple binding modes with NoV [4,5]. Structural analysis of the binding of NoV GII.4 virus-like particles (VLPs) to A-, B-, H-, and Lewis-type HBGAs by X-ray crystallography showed the presence of a fucose binding site in the dimer formed by polymerization of the P-structural domains of the VLPs, which recognizes the HBGAs and binds to them via hydrogen bonding [5,6]. Several HBGA-like molecules are present in oysters, which specifically bind to NoV and bioaccumulate [7].

Fucoidans have been extensively studied for a range of health benefits, including anti-inflammatory, hypolipidemic and antithrombotic properties [8]. In addition, the antiviral effect of fucoidans has been demonstrated in herpes simplex virus, cytomegalovirus and human immunodeficiency virus [9]. It has also been reported that *Laminaria japonica* fucoidan (LJ fucoidan) has a significant inhibitory effect on NoV, but there are few reports on the inhibition of NoV by SFP [10].

In the present study, we isolated and purified five polysaccharides from *Sargassum fusiforme*, labeled pSFP-1-5. The results showed that pSFP-5 had the best anti-NoV GII.4 activity and could reduce the risk of NoV transmission.

## Materials and methods

### Isolation, purification, and classification of SFP

The *S. fusiform* used in the experiments was provided by Wenzhou Dongtou Sargassum fusiform farming base and was purified using a previously reported method [11]. In short, SFP powder was first dissolved at a polysaccharide-to-water ratio of 1:10 and mixed with 4 M CaCl_2_ to obtain a transparent supernatant by centrifugation (4,000 × ***g***, 15 min). The supernatant was dialyzed and concentrated with 95% ethanol. The concentrated solution was standing (4°C, 12 h) and centrifuged (10,000 × ***g***, 15 min). The precipitate was dried to obtain SFP, which was then dissolved according to the aforementioned ratio (1:10). The protein was removed by the Sevage method to obtain crude SFP. The partial SFP (pSFP) was prepared as the solution (0.1 g/mL) and then passed through a 0.22 µm filter membrane to remove other insoluble impurities. Finally, the pSFP solution was eluted using 0, 0.1, 0.3, 0.5, and 1 M NaCl solutions through a DEAE cellulose column (2.5 × 50 cm), and the fractions were collected using a polysaccharide purifier. Subsequently, the absorbance values of the collected fractions were measured at OD_490nm_ according to the phenol-sulfuric acid method, and mixed the same fractions to obtain five SFPs labelled pSFP-1-5.

### The physicochemical characterization of pSFP-1-5

The total sugar was determined by the phenol-sulfuric acid method [12]. Fucoidan content was determined by the methylene blue method [13]. Sulfate content was determined by the turbidimetric method, and glyoxylate content was determined by the m-hydroxydiphenyl method [14,15]. Molecular weight was determined by high-performance gel permeation chromatography [16]. The monosaccharide composition was determined by the 1-phenyl-3-methyl-5-pyrazolinone (PMP) derivatization method [17]. Finally, fourier transform infrared spectroscopy (FTIR) analysis was performed.

### Handling of saliva and Identification of blood group antigens

Our 120 saliva samples were generously gifted by the Chinese Center for Disease Control and Prevention, including 93 samples of known ABO blood group [18]. The treatment of saliva was modified according to the existing research [19]. The saliva samples were boiled at 100°C for 10 min and then centrifuged (10,000 × ***g***, 10 min) to obtain a clear supernatant. Saliva blood group antigens were determined according to the protocol described in the reference, with slight modifications to test for A, B, and O blood group antigens [20]. The saliva samples were diluted 1:100 with phosphate-buffered saline (PBS) and 100 µl/well of the diluted samples were added to a 96-well enzyme plate. The plate was incubated at 4°C for 12 h. After washing with PBS, the plate was blocked with 10% skimmed milk powder (Inner, 300 µl/well). After incubation at 37°C for 2 h, the blocking solution was discarded and washed with PBS for five times. Approximately 100 µl of monoclonal antibodies (BG-2, BG-3, and BG-4; BioLegend, San Diego, CA, U.S.A.) were added to the blood group antigen HBGAs, diluted to 1:1,000, added to the plate, and incubated at 37 °C for 1 h. The plate was washed as above. Next, TMB substrate (3,3',5,5'-tetramethylbenzidine; Sigma Aldrich,100 µl/well) was added to the plate and the reaction proceeded for 20 min at room temperature in the dark. The OD_450nm_ value was measured using a multifunctional enzyme marker (BioTek, Epoch, U.S.A.). The negative control group OD_450nm_ value was ≤ 0.1. A *P/N* value > 2.0 was used as the standard for determining positivity.

### Determination of the activity of pSFP against NoV GII.4 VLPs

Anti-NoV GII.4 VLPs activity of pSFP fractions was determined according to a previously reported method [21]. The results were expressed as inhibition rate, calculated as:

$$\mathrm{Inhibition}\%=\left(1-\frac{Sample\;{OD}_{450}}{Positive\;control\;{OD}_{450}}\right)\times 100\%\;\;$$

### Inhibition of NoV GII.4 VLPs binding to oyster HBGA-like molecules by pSFP-5

Eighty oysters were dissected to extract the digestive tracts. After weighing, 400 μl PBS was added and centrifuged (10,000 × ***g***, 3 min), and then 95°C water bath for 10 min. Thereafter, it was centrifuged (10,000× ***g***, 10 min) at 4°C to obtain the supernatant. The supernatant was added to a 96-well enzyme standard plate (100 μl/well) with PBS as a negative control, and incubated overnight at 4°C. Next, oyster HBGA-like molecules of type A, B, and H were screened, and the inhibitory effect of pSFP-5 on the binding of NoV GII.4 VLPs to oyster HBGA-like molecules was determined. The results were expressed as inhibition rate.

### Inhibiting the accumulation of NoV GII.4 in oysters using pSFP-5

NoV GII.4 positive fecal samples were provided by the Chinese Center for Disease Control and Prevention. The virus fecal samples were diluted with PBS and centrifuged (10,000 × ***g***, 5 min) at room temperature. NoV RNA was extracted from the virus diluted suspension using a TIANamp Virus RNA Kit (TIANGEN, Beijing, China) and real-time quantitative analysis was performed on a LightCycler® 96 Real-Time PCR instrument (Roche Diagnostics, Risch-Rotkreuz, Switzerland). The cycling conditions were 42°C for 15 min and 95°C for 2 min. Then 95°C 5 s, 56°C 30 s, 37°C 5 min, a total of 45 cycles. Primers and probe sequences are shown in Table 1.

**Table 1:** Sequences of primers and probe

Name	Sequence (5′ → 3′)
Forward primer	ATGTTCAGRTGGATGAGRTTCTCWGA
Reverse primer	TCGACGCCATCTTCATTCACA
Probe	FAM-AGCACGTGGGAGGGCGATCG-TAMRA

The oyster samples were vigorous adult oysters purchased from Wenzhou seafood market. Five experimental groups were set up, namely negative control group, positive control group, high pSFP-5 group (12 mg/ml), medium pSFP-5 group (6 mg/ml), and low pSFP-5 group (3 mg/ml), corresponding to 1-5 beakers respectively. Approximately 0.5 L seawater was added to each beaker, after which 50 μl of the virus (2.86 × 10^10^ copies/μl) was added to beakers 2-5. Then, pSFP-5 was added to beakers 3-5 at 6, 3, and 1.5 g, respectively, and all beakers were left at 25°C with oxygen being passed for 24 h. Then oysters were removed, rinsed with sterile distilled water, dried, and transferred to aseptic conditions. Five oysters from each group were opened with a sterile knife and the digestive tracts were removed. Next, the digestive tracts were added to 300 µl RNA enzyme-free water and ground for 15 min, after which 5 ml proteinase K (100 μg/ml) solution was added, and the mixture was sequentially incubated for 60 min at 37°C and 15 min at 60 ± 2°C. Subsequently, the mixture was centrifuged (10,000 × ***g***, 10 min) at 4°C to obtain the supernatant, and NaCl was added to a final concentration of 0.5 M [22]. Next, PEG 6000 was added in equal amounts to a final concentration of 10% and overnight at 4°C, then centrifuged (10,000 × ***g***, 30 min) at 4°C to collect the virus precipitate and dissolved in PBS [23].

The experiment consisted of five groups (Table 2). Each group was incubated at 37°C for 1 h at a volume ratio of 1:1. Next, the treated viral proteins were added dropwise onto a carbon-covered copper mesh membrane, and the samples were adsorbed for 3-5 min and then blotted off with filter paper. Finally, 1 drop of 2% phosphotungstic acid was added for 2 min for staining, after which the staining solution was aspirated, and the copper mesh was dried at room temperature. The NoV GII.4 VLPs were observed using TEM with an accelerating voltage of 200 kV, and viral protein images were obtained with a CCD camera system (50 k magnification) [24,25].

**Table 2:** The experimental group of NoV GII.4 VLPs treated with pSFP-5

Number	Experimental group composition
1	NoV GII.4 VLPs (100 μg/mL), deionized water
2	NoV GII.4 VLPs (100 μg/mL), type A HBGA-like molecules
3	NoV GII.4 VLPs (100 μg/mL), type A HBGA-like molecules, pSFP-5 (3 mg/ml)
4	NoV GII.4 VLPs (100 μg/mL), type H HBGA-like molecules
5	NoV GII.4 VLPs (100 μg/mL), type H HBGA-like molecules, pSFP-5 (3 mg/ml)

### Statistical Analysis

Statistical analysis was performed using GraphPad Prism 8 (GraphPad Software, San Diego, CA, U.S.A.). One-way ANOVA, unpaired two-tailed* t*-test and Tukey multiple comparisons were used to analyze the statistical differences between the groups. *P*<0.05 for a significant difference, *P*<0.01 for a very significant difference.

## Results

### Classification and purification of SFP

**Figure 1:**
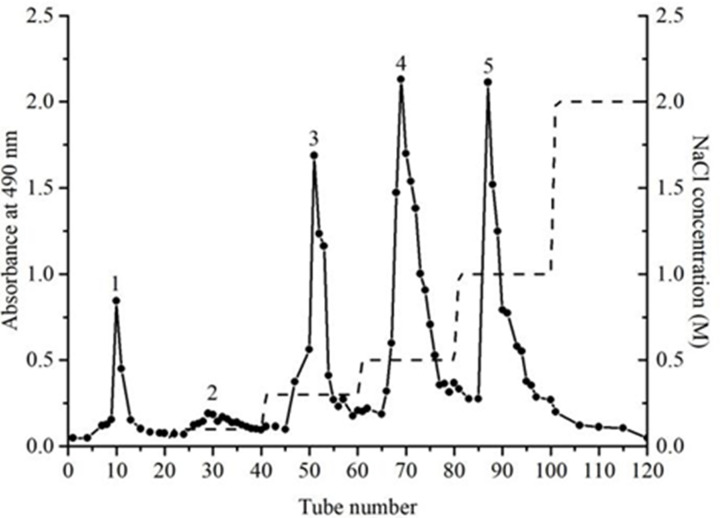
Separation of *S. fusiform* fucoidan using DEAE cellulose ion chromatography 1, 2, 3, 4, and 5 were the fractions eluted from 0, 0.1, 0.3, 0.5, and 1 M NaCl solutions, respectively.

The crude SFP usually contains many impurities, and the impurities such as free protein can affect the structural identification, purity and activity of the polysaccharide. Therefore, DEAE cellulose ion chromatography was used to classify and purify the crude SFP. The result is shown in Figure 1. Five main components were collected according to the elution curve and named pSFP-1, pSFP-2, pSFP-3, pSFP-4, and pSFP-5, respectively.

### Chemical composition and monosaccharide composition of pSFP-1-5

As shown in Table 3, the polysaccharide content of pSFP-1-5 ranged from 33.37% to 42.24%. The Fucoidan content of pSFP-1-5 ranged from 10.64% to 11.86%. pSFP-5 had higher contents of glyoxylate and sulphate at 27.08% and 16.08%, respectively. The molecular weight results showed that pSFP-1 and pSFP-2 had higher molecular weights at 58.07 and 55.65 kDa, respectively, while pSFP-5 had the lowest molecular weight at 21.33 kDa. Table 4 showed the results of the monosaccharide composition of the five polysaccharides. The results showed that pSFP-1-5 were all complex polysaccharides, consisting mainly of mannose, rhamnose, glucuronic acid, glucose, galactose, xylose and fucose. In addition to the four basic components of mannose, rhamnose, glucuronic acid and fucose, there were some variations in the monosaccharide composition of the five polysaccharides. For example, the monosaccharide composition of pSFP-1, pSFP-2 and pSFP-5 also contained galactose and those of pSFP-3 and pSFP-4 also included glucose. The monosaccharide composition of pSFP-5 was the simplest among all components, containing only five monosaccharides.

**Table 3:** Chemical composition of pSFP-1-5

	pSFP-1	pSFP-2	pSFP-3	pSFP-4	pSFP-5
Total sugar content (%)	42.24 ± 0.35	36.15 ± 0.23	33.37 ± 0.93	35.33 ± 0.61	38.15 ± 0.73
Fucoidan content (%)	11.33 ± 0.64	11.03 ± 1.05	10.64 ± 0.61	10.84 ± 0.37	11.86 ± 1.17
Glyoxylate content (%)	30.51 ± 0.77	24.86 ± 1.26	22.28 ± 0.70	27.77 ± 0.48	27.08 ± 0.70
Sulfate content (%)	7.31 ± 0.14	7.7 2± 0.05	16.53 ± 0.14	14.50 ± 0.58	16.08 ± 0.48
Molecular weight (kDa)	58.07 ± 0.17	55.65 ± 0.07	44.43 ± 0.03	22.61 ± 0.03	21.33 ± 0.31

±: standard deviation.

**Table 4:** Monosaccharide composition of pSFP-1-5

Samples	Monosaccharide composition						
	Man	Rha	GlcUA	Glc	Gal	Xyl	Fuc
pSFP-1	0.31	0.40	0.52	nd	0.82	0.85	1.00
pSFP-2	0.31	0.40	0.52	nd	0.81	0.84	1.00
pSFP-3	0.31	0.40	0.52	0.70	nd	0.85	1.00
pSFP-4	0.31	0.40	0.53	0.70	nd	0.85	1.00
pSFP-5	0.31	0.40	0.53	nd	0.82	nd	1.00

nd: not detected.

**Figure 2:**
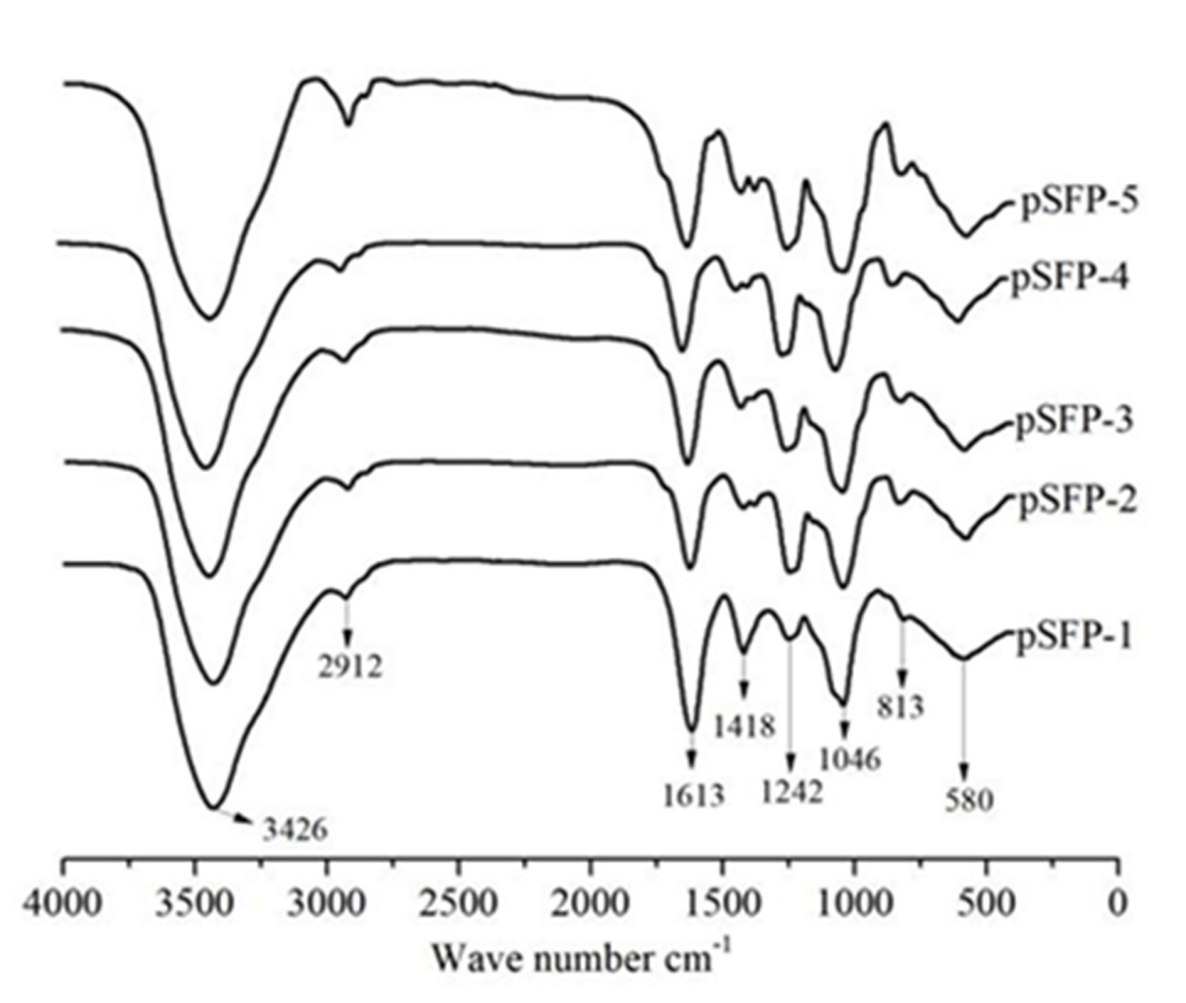
FTIR spectra of the pSFP-1-5 polysaccharide fractions

As shown in Figure 2, the absorption peaks of the five polysaccharides were similar, indicating that the main organic functional groups of the five polysaccharides were similar. All fractions had absorption peaks at 3426 and 2912 cm^−1^, with the wide peak at 3426 cm^−1^ being caused by the stretching vibration of O-H on the sugar ring, and the peak at 2912 cm^−1^ corresponded to the C-H expansion and contraction signal [26]. The absorption peak at 1613 cm^−1^ was related to the stretching vibration of carboxylic acid C=O. The stretching vibration peak at 1242 cm^−1^ corresponded to S=O, indicating that the polysaccharides contained sulfate groups. The absorption peak at 1046 cm^−1^ was the characteristic peak of C-O-C, and the absorption peak at 813 cm^−1^ was the characteristic peak of a β-glycosidic bond, indicating that the five polysaccharides contain β-glycosidic bonding.

### Inhibition of NoV GII.4 VLPs binding to A, B, and H HBGAs by pSFP-5

Since the type A saliva has the highest HBGA content, and type A HBGAs bind best to NoV GI and GII [27,28], we firstly screened for the optimal anti-NoV activity level using the inhibition rate of type A HBGAs binding to NoV GII.4 VLPs. Preliminary optimization experiments determined that when the concentration of pSFP-1-5 was 3 mg/ml, each component showed the strongest anti-NoV activity.

**Figure 3:**
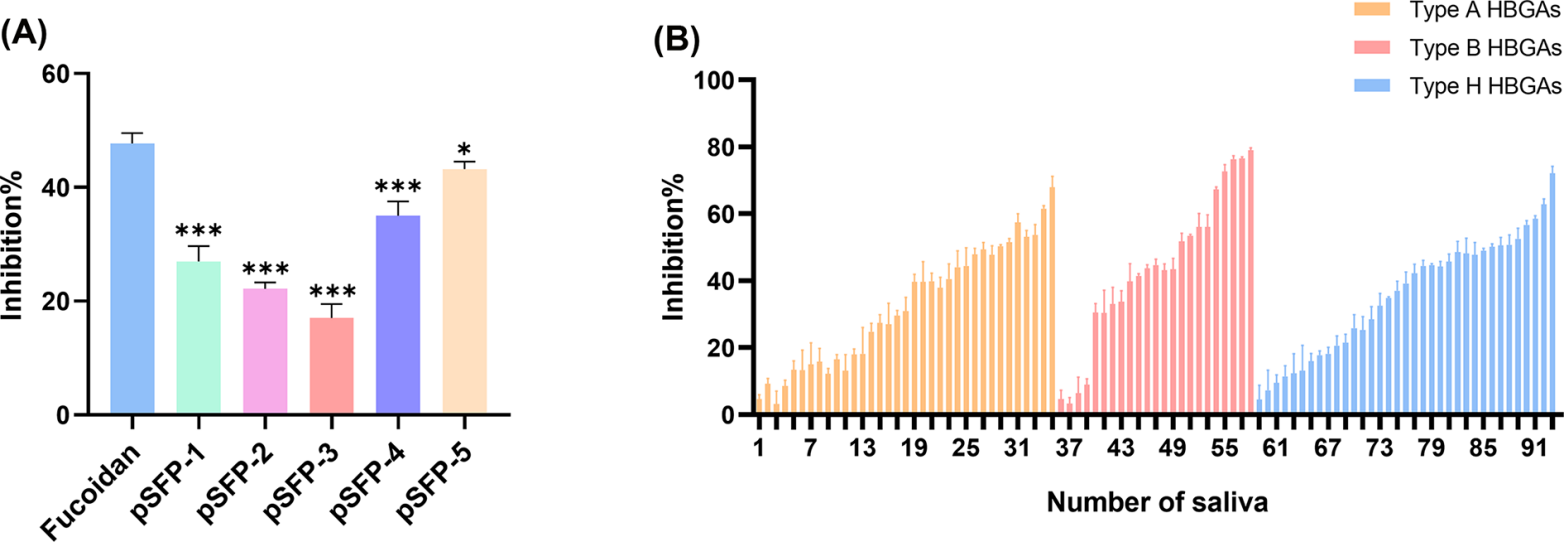
Inhibition of NoV GII.4 VLPs binding to A, B, and H HBGAs by pSFP-5 (**A**) Activity of pSFP against NoV GII.4 VLPs binding to HBGAs at 3 mg/ml level. pSFP-1-5 were all compared with Fucoidan. Fucoidan was the fucoidan standard sample. Data were expressed as mean ± standard deviation. *P<0.05, ***P<0.001. (**B**) The activity of pSFP-5 against NoV GII.4 VLPs binding to type A, B and H HBGAs. A total of 93 saliva samples were tested for antiviral activity. X-axis indicated the number of saliva samples under type A, B, H HBGAs. Error bars indicated standard deviation.

As illustrated in Figure 3A, the effect of pSFP-1-5 (3 mg/ml) on the binding of type A HBGAs to NoV GII.4 VLPs was evaluated by ELISA. Among the five components, pSFP-3 showed the least effective in inhibiting the binding of type A HBGAs to NoV GII.4 VLPs, whereas pSFP-5 showed the best anti-NoV GII.4 VLPs activity, which might be due to it having the smallest molecular weight and thus is more conducive to the exposure of structures that function to inhibit viral activity [29]. In addition, pSFP-5 was the most abundant and readily available, and its fucoidan and sulfate contents were also higher. Therefore, we chose pSFP-5 for subsequent experiments.

In addition, pSFP-5 could also inhibit the binding of type B and H HBGAs to NoV GII.4 VLPs, and the inhibition effect on type B HBGAs was better, up to 78% (Figure 3B).

### Inhibition of oyster HBGA-like molecules binding to NoV GII.4 VLPs by pSFP-5

**Figure 4:**
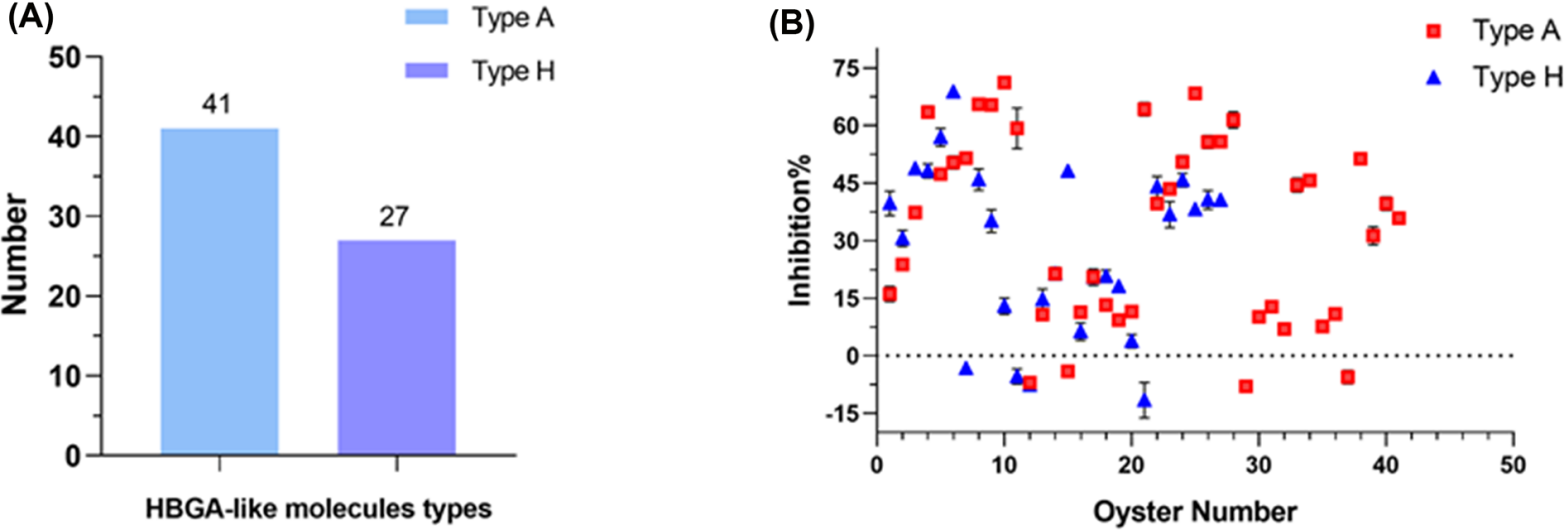
Identification of HBGAs in the digestive tract of oysters and the activity of pSFP-5 against NoV GII.4 VLPs binding to A and H HBGAs (**A**) Identification of HBGA-like molecules in the oyster digestive tract using ELISA. (**B**) The activity of pSFP-5 against NoV GII.4 VLPs binding to A and H HBGAs. The X-axis represented a single oyster under each number (Type A, No.1-41; Type H, No.1-27). Error bars indicated standard deviation.

Oysters can accumulate a large amount of NoV in their bodies and are important carriers of NoV transmission [30]. To explore the inhibitory effect of pSFP-5 on the binding of HBGA-like molecules to NoV GII.4 VLPs in oysters, we firstly characterized the HBGA-like molecule types in 68 oysters digestive tracts using ELISA (Figure 4A). More oysters contained type A HBGA-like molecules, which was consistent with the report that NoV GI, which bound to type A HBGA-like molecules, became the dominant strain of NoV infection in oysters [31]. Whereafter, we determined the *in vitro* inhibitory activity of pSFP-5 against NoV GII.4 VLPs in these 68 oysters (Figure 4B). The result showed that pSFP-5 inhibited the binding of most HBGA-like molecules (88%) to NoV GII.4 VLPs. However, in individual oysters, pSFP-5 did not show an inhibitory effect on the combination of the two, which may be due to the presence of ingredients in these oysters that prevented pSFP-5 from exerting its effect.

### Inhibition of NoV GII.4 accumulation in oysters by pSFP-5

**Figure 5:**
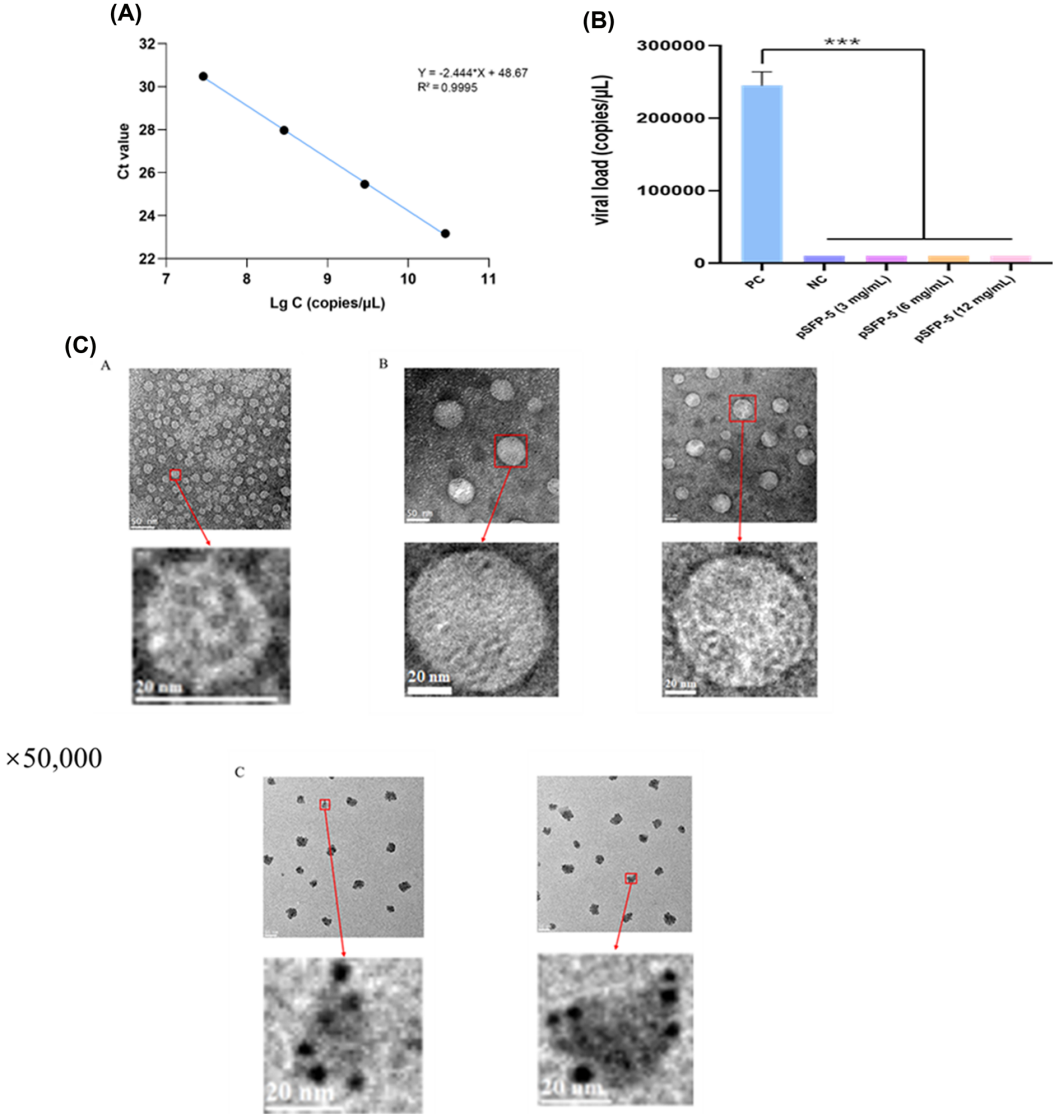
pSFP-5 inhibited NoV GII.4 accumulation (**A**) Standard curve of RT-qPCR. The X-axis was Log_10_(copy number), C represented copy number. (**B**) Determination of viral load. PC, positive control. NC, negative control. Error bars indicated standard deviation. ***P<0.001. (**C**) NoV GII.4 VLPs observed by TEM. (A) NoV GII.4 VLPs were treated with deionized water and locally magnified. (B) NoV GII.4 VLPs bound to oyster type A and H HBGA-like molecules and locally magnified. (C) NoV GII.4 VLPs were treated with pSFP-5 after binding to oyster type A and H HBGA-like molecules and locally magnified.

Bivalve mollusks are an important source of foodborne diseases. NoV is readily enriched and difficult to excrete from bivalve mollusks, which means that the consumption of bivalve mollusks often leads to gastrointestinal diseases in humans [32]. Therefore, inhibiting NoV accumulation in oysters is essential to reduce the risk of foodborne diseases from seafood. We gradiently diluted the virus samples and quantified them by RT-qPCR. As shown in Figure 5A, there was a good linear relationship between the Ct value and the virus copy number. Subsequently, we measured the virus content in oysters (Figure 5B). The results showed that no virus was detected in both the NC group and the experimental groups treated with pSFP-5, indicating that pSFP-5 inhibited the accumulation of NoV in oysters and had a significant inhibitory effect at a concentration of only 3 mg/ml.

To further explain that pSFP-5 could prevent the accumulation of NoV in oysters, we used TEM to observe the morphological changes of NoV GII.4 VLPs after pSFP-5 treatment (Figure 5C). A showed that NoV GII.4 VLPs were spherical, the shape of the particles was intact, and the diameter was approximately 25–30 nm. B indicated that NoV GII.4 VLPs presented a spherical structure after combination with oyster HBGA-like molecules, and the shape became significantly larger, with a diameter of approximately 50–80 nm. This may lead to a more stable state of the virus particles, which is conducive to biological accumulation in oysters. As demonstrated in C, after pSFP-5 treatment, the spherical appearance of NoV GII.4 VLPs changed significantly, showing an irregular shape. This change may lead to instability or even loss of binding ability between NoV GII.4 VLPs and oyster HBGA-like molecules, thereby reducing the accumulation in oysters. Therefore, pSFP-5 may reduce the affinity of NoV with oyster HBGA-like molecules by causing NoV to deform to achieve antiviral effects.

## Discussion

NoV has strong environmental resistance, low infection dose and short incubation period, and the whole population is generally susceptible. At present, there is no specific drug, so the study of anti-NoV substances is very necessary. NoV binds to HBGAs through capsid proteins to attach to host cells and cause infection [33–35]. Therefore, interrupting the binding of NoV to HBGAs may be an effective strategy to prevent NoV infection. Plant extracts have natural economic benefits and safety in preventing and treating viral infections, but there are few studies on the anti-NoV activity of plant extracts [36].

Fucoidan is a substance extracted from brown algae, which has a wide range of immunomodulatory, tumor cell growth inhibition, and antiviral biological activity [37,38]. In this study, fucoidan was extracted and purified from *S*. *fusiforme*, and five polysaccharide fractions were obtained, namely pSFP-1-5. pSFP-5, the best component, was screened by the inhibition rate of type A HBGAs and NoV GII.4 VLPs. It may be because the molecular weight of pSFP-5 is the smallest, which is conducive to expose the structures that inhibit viral activity. At the same time, the extraction rate of pSFP-5 is high and easy to obtain. Therefore, pSFP-5 was selected for subsequent experiments. Further studies have found that pSFP-5 can also significantly inhibit the binding of type B and H HBGAs to viral particles.

In view of the fact that high-temperature cooking affects the freshness of oysters, simple cooking or direct raw eating of oysters is a method adopted by many people. This method does little to reduce NoV accumulation in oysters, making eating oysters an important way to infect NoV. In this study, oysters were artificially contaminated and treated with pSFP-5. Finally, no virus was detected in the digestive tract tissues of oysters, suggesting that pSFP-5 effectively reduced the accumulation of NoV in oysters. The results of electron microscopy showed that the virus particles were severely deformed after pSFP-5 treatment, which may cause the virus particles to fail to bind to HBGAs correctly, thereby achieving a good inhibitory effect. The results of Lim et al.also showed that the deformation of virus particles is a new strategy to make the virus lose its infectivity [39].

According to our research, pSFP-5 shows a good application prospect in anti-NoV. The reason why pSFP-5 exerts antiviral activity may be that the morphology of virus particles changes significantly and cannot bind to HBGAs, but the specific mechanism of action needs further study. In addition, the new method of polysaccharide separation and purification may reveal more significant differences among the five components, and provide stronger support for the establishment of the best component.

## Data Availability

The datasets generated during and/or analysed during the current study are available from the corresponding author on reasonable request

## Abbreviations

HBGA: histo-blood group antigen
NoV: norovirus
ORF: open reading frame
VLP: virus-like particle
